# GEOGRAPHIC PATTERNS IN THE LOCATIONS OF ADULT DAY CENTERS AND NURSING HOMES IN TEXAS

**DOI:** 10.1093/geroni/igae098.0076

**Published:** 2024-12-31

**Authors:** Brian Downer

**Affiliations:** The University of Texas Medical Branch, Galveston, Texas, United States

## Abstract

Most older adults want to live at home in a community setting and avoid moving to a nursing home. Adult day centers (ADCs) are a valuable resource that can help older adults live at home. However, a concern is older adults may not have access to ADCs to benefit from their services. This analysis describes geographic patterns in the locations of ADCs and nursing homes in Texas. We used data from the Texas Department of Health and Human Services and the Centers for Medicare and Medicaid Services (CMS) to identify all licensed ADCs and CMS certified nursing homes, respectively, in Texas. Data for county characteristics (population, % aged >65) came from the US Census. Texas has 425 ADCs and 1,195 nursing homes. Among the 254 Texas counties, 48 (18.9%) have at least one ADC and 217 (85.4%) have at least one nursing home. Of the 37 counties with no nursing home, 1 county had an ADC while 170 of the 206 counties without an ADC had at least one nursing home. Approximately 6% (n=73) of nursing homes and 54.8% of ADCs (n=233) were in the thirteen counties boarding Mexico, with Hidalgo County (total population n=855,175; 11.5% aged >65) having nearly one-third of all ADCs (n=132). Conversely, the five most populous counties in Texas (total population n=12,482,916; 12.0% aged >65) had 26.1% of ADCs (n=111) and 30.0% of nursing homes (n=354). These findings suggest that older adults living in large metropolitan areas of Texas may have inadequate access to ADCs.

